# Hospitalisation Cost of Patients with Diabetic Foot Ulcers in Valencia (Spain) in the Period 2009–2013: A Retrospective Descriptive Analysis

**DOI:** 10.3390/ijerph15091831

**Published:** 2018-08-24

**Authors:** Pilar Nieto-Gil, Ana Belen Ortega-Avila, Manuel Pardo-Rios, Manuel Cobo-Najar, Carlos Blasco-Garcia, Gabriel Gijon-Nogueron

**Affiliations:** 1 Department of Nursing and Podiatry, University of Valencia, 46010 Valencia, Spain; m.pilar.nieto@uv.es (P.N.-G.); carlos.blasco@uv.es (C.B.-G.); 2 Department of Nursing and Podiatry, University of Malaga, IBIMA, 29071 Malaga, Spain; gagijon@uma.es; 3 Department of Podiatry, Catholic University of Murcia, 30107 Murcia, Spain; mpardo@ucam.edu (M.P.-R.); familiacobofrutos@gmail.com (M.C.-N.)

**Keywords:** diabetic foot, ulcers, costs, MBDS (Minimum Basic Data Set)

## Abstract

Ulcers are the main cause of hospitalisation and clinical complications in patients with diabetes. We analyse the length and cost of hospital stay of patients with diabetic foot ulcers, taking into consideration that hospitalisation and, if necessary, amputation represent the greatest area of expense to the healthcare system for such patients. This analysis focuses on the treatment provided to these patients in public hospitals in the region of Valencia (Spain), registered in the Spanish Minimum Basic Data Set, during the period 2009–2013. The number of acute hospital admissions in this respect is increasing and has a high socioeconomic cost. During the study period, there were over 2700 hospital admissions, an average of nearly 550 per year. The total hospital stay for these patients was 30,886 days, with an average of 11.4 days and a cost of €7633 per admission. Preventive policies and the deployment of multidisciplinary teams are essential to reduce these costs and avoid future complications such as amputation.

## 1. Introduction

The 2007 International Consensus on the Diabetic Foot, edited by the International Working Group on the Diabetic Foot, defines diabetic foot (DF) as the infection, ulceration and destruction of deep tissues, associated with neurological abnormalities and peripheral vasculopathy of diverse severity in the lower extremities, as a result of the interaction of factors induced by sustained and uncontrolled hyperglycaemia.

The most common cause of complications and hospitalisation in diabetic patients are those associated with DF. Approximately 15% of diabetic patients will develop a foot or leg ulcer during their illness [1]. Of these cases, 85% will ultimately require amputation, an outcome that represents 40–60% of all non-trauma hospital amputations [2].

DF ulcer is a clinical alteration of neuropathic aetiopathogenic origin, arising from sustained hyperglycaemia, with or without concurrent ischaemia and/or previous traumatic trigger, injury to and/or ulceration of the foot [3]. Depending on the aetiological factors involved, ulcers may be neuropathic (55%), ischaemic (10%) or neuroischaemic (35%) [4]. The presence of diabetes increases the risk of ulceration and amputation by 25% [5]. DF ulcers mainly affect patients aged 45–65 years, who present an incidence of 15% [6].

The economic impact of DF is very significant. Globally, it is estimated that 11% of total health expenditure is dedicated to meeting the needs of diabetic patients [7]. In the European Union, the estimated average cost per patient with type 2 diabetes mellitus (DM-2) is €2834 per year, and the average duration of hospitalisation is 23 days per year [8]. The Morales Meseguer Hospital in Murcia (SE Spain) used the Minimum Basic Data Set (MBDS) to study patients with DF and reported high levels of mortality and an average hospital stay of 11 days [9].

In the United States, DF is estimated to cost about $5500 per patient per year, at two years after the onset of illness, although this figure may reach $28,000 if amputation is required [10]. In Europe, the 2008 Eurodiale study reported the direct cost of a foot ulcer that healed to be €7147, but if no healing was achieved in 12 months, the cost rose to €18,790; if amputation was necessary the hospital cost was €24,540 [11]. In Austria, a 2007 study by Habacher et al. reported the cost of non-diabetic patients to be €1071 compared to the €7844 incurred for patients with peripheral vascular disease and infection [12].

The main aim of the present study is to analyse the length and cost of hospital stay for patients with DF ulcers, taking into account that hospitalisation and, if needed, amputation are the most significant areas of healthcare expenditure for patients with DF [13,14,15]. The second study aim is to determine the influence of age, gender and type of admission on the cost of hospitalisation for DF patients.

## 2. Method

### 2.1. Design

In this retrospective, descriptive, ecological and observational study, the MBDS was analysed with respect to the period 1 January 2009 to 31 December 2013, to extract statistical data related to patients with DF admitted to public acute care hospitals in the region of Valencia (Spain). The statistics for patients with chronic conditions were excluded from this analysis. The study protocol was approved by the Medical Research Ethics Committee of the University of Malaga (CEUMA 2015-026-H).

### 2.2. Subjects

The patients included in the study all presented DF ulcers. The admission and treatment cost of any comorbidities was not included in the analysis of their hospitalisation. The sample is a probabilistic population.

The inclusion criteria were hospital discharge codes 250.x and, in some secondary diagnoses, 707.x (except pressure ulcers). We also searched for patients coded with 707.x (except pressure ulcers) as the main diagnosis and 250.x in secondary diagnostic fields. The study population was obtained as the outcome of the two searches. Code 250.xx, the diagnosis of diabetes mellitus code 707.xx was further broken down, into the following sub-codes: 707.10, 707.13, 707.14, 707.15 and 707.19, all of which included foot ulcers, except any pressure ulcers.

### 2.3. Procedure

The following study variables were considered: hospital admission, gender, age, DRG (diagnostic related group), MDG (major diagnostic group), procedure.

The MBDS is a hospital IT database, published annually to reflect the case mix of patients treated. The system records the number and type of patients and the human and material resources used. The data are recorded when the patient is discharged from hospital, and the following fields are included in the database:

- Patient identification: clinical history number, date of birth and sex;
- Identification of the episode: date of admission, date of registration, admission service and discharge service;
- Clinical data: main diagnosis, secondary diagnosis and procedure.

Each MBDS-coded discharge is associated with a DRG code. In the present study, this code, in conjunction with the Valencia Government Schedule of Clinical Expenses, was used to calculate the cost of each hospital admission. In the present study, we analysed the evolution of the changes in these costs during the study period. The relevant legislative documents considered were the general provisions with respect to Valencia in the editions of the Official Spanish Gazette (BOE) published on 31 January 2009, 27 January 2011, 27 January 2012 and 24 January 2013. DRG was stable 2009–2012, but in 2013 increased by 0.5 point. The official rates for hospital care, including all the health services provided at a given location for a hospitalised patient, are calculated according to their complexity and the human and material resources consumed. 

DRG is a patient classification system that enables the analyst to relate clinical conditions to the cost of health care. Patients are grouped according to the severity of their disease, the prognosis, the need for treatment, its difficulty and the resources consumed in this process. Each DRG is assigned a relative weight, which represents the expected cost of this hospital admission in relation to the average hospitalisation cost of an acute hospital patient. Thus, an analysis can be performed taking into account the complexity of the hospital admission and the human and material resources required. The relative weights are listed on the website of the Spanish Health Ministry (www.msssi.gob.es).

### 2.4. Statistical Analysis

The MBDS data were received as Microsoft Access 2007 and Microsoft Excel 2007 files and then analysed using IBM SPSS v.20 statistical software (SPSS Inc., Chicago, IL, USA).

The data were first subjected to a frequency analysis, to obtain the number of cases and their percentages, and then descriptively enhanced, by age, sex, hospital and department. The descriptive results are shown as the frequency, percentage and cumulative percentage. For the continuous variables (hospital costs and length of stay), the means and standard deviations were calculated.

The chi-square test was used to compare the differences between the categorical variables: age, sex, hospital and department, and the distributions, such as hospital admissions and discharges.

For hospital costs and length of stay, the differences by year, sex, age, hospital and department were compared by one-way ANOVA (Test *F*). The Tamhane post hoc test was applied when the homogeneity tests revealed significant variances, and the least significant difference (LSD) post hoc test when there were no significant variances. The threshold of statistical significance was taken as *p* > 0.05.

## 3. Results

**Figure 1:**
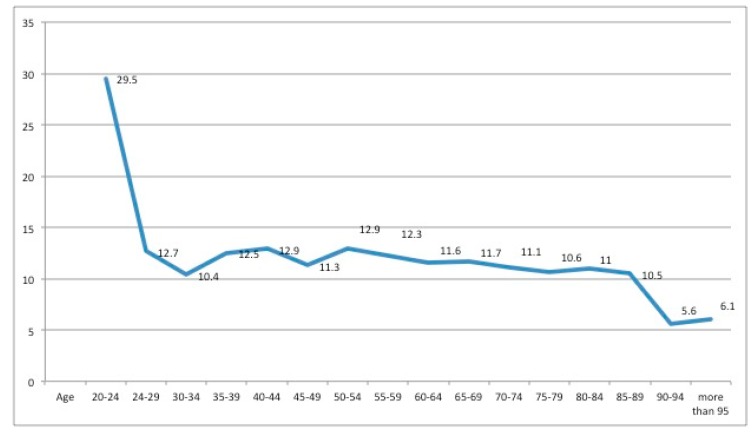
Average length of hospital stay (days), by age.

During the study period, 2702 hospital discharges were recorded for patients with DF ulcers from acute-care hospitals in Valencia, an average of 547.6 discharges per year (SD 26.39). The total hospital stay for this condition was 30,886 days. The mean length of stay was 11.4 days per admission (SD 12.4), and the range was 1–234 days (Figure 1).

More than twice as many men as women were admitted to hospital with DF ulcers (1868 vs. 834; *p* < 0.000). (Similarly, the total length of hospital stay was much greater for men, at 21,163 days vs. 9723 days for women.) However, the average stay was evenly matched between the sexes, at 11.3 days (SD 11.7) and 11.7 days (SD 13.9) for men and women, respectively, with no significant differences (*F* = 0.406, *p* = 0.524).

By age groups, the mean length of hospital stay ranged from 10.4 days by patients aged 30–34 years to 12.9 days by those aged 50–54 years. The largest number of hospital admissions was by patients aged 75–79 years, with an average stay of 10.6 days. Strikingly, the patients aged 20–24 years presented an average stay of 29.5 days, while those at the other end of the scale, aged 90–94 years and 95 or more, required stays of only 5.6 and 6.1 days, respectively. Nevertheless, these youngest and oldest patients were relatively infrequent, and the ANOVA did not reveal significant differences.

During the study period, the total hospital cost of providing treatment to patients with DF ulcers was €20,624,337, an average of €7633 per patient (SD 4.189) (Table 1).

The total hospital cost decreased progressively from 2009 to 2012, but rebounded in 2013. ANOVA revealed significant differences (*F* = 2.407, *p* = 0.047) between 2010 and 2013 and between 2011 and 2013. Hospital admission rates were similar during 2009, 2010 and 2011, but the BOE assigned less complicated cases to this DRG (Table 2), hence a lower relative weight and hence higher costs. The cost changes observed over the study period may also be related to the four adjustments made to the official rates in 2009, 2011, 2012 and 2013, which were minimal or null (range 0.15).

During the study period, the total hospital cost for male patients was €14,386,532, an average of €7702 per case. The corresponding costs for female patients were €6,237,805 and €7479. The large difference in total cost was due to the number of cases treated, because the average costs did not present significant differences (*F* = 1629, *p* = 0.202) (Table 3).

The age group presenting the highest number of admissions was that of patients aged 75–79 years (*n* = 364), producing a total cost of €2,721,309, which was also the highest total cost.

However, the highest average cost was produced by the patients aged 55–59 years (*n* = 282), at €8059. The lowest average cost corresponded to those aged under 39 years (*n* = 48), at €6714, but this group did not account for the lowest overall cost, which was produced by the patients aged over 90 years (*n* = 46), with €31,872. Nevertheless, the mean costs by patient age group did not present statistically significant differences (*F* = 1.182, *p* = 0.294). By sex and age group, the highest total cost during the study period was produced by men aged 65–69 years, with €2,045,971, followed by those aged 60–64 years, with €1,926,964. Among the female patients, the highest overall cost was produced by those aged 80–84 years, with €1,096,037, followed by the group aged 75–79 years, with €1,093,499 (Table 4).

## 4. Discussion

During the five years analysed, 2702 hospital admissions of patients with DF ulcers were recorded, generating 30,886 days of stay, with a mean of 11.4 days per case attended. This finding is in line with a previous study carried out at the Moral Meseguer Hospital (Murcia, Spain), according to which the average hospital stay for these patients was 11 days [9]. In a Europe-wide study, the corresponding rate has been estimated at 23 days [8].

Analysis by sex revealed significant differences in the length and cost of hospital stay. Thus, male patients required a total of 21,163 days of stay compared to the 9723 days for females. The mean length of hospital stay was 11.3 and 11.7 days, respectively. In financial terms, male patients produced an average cost of €7701 compared to €7479 by female patients. However, significant differences were observed in the total cost, which was €14,386,532 for men and only €6,237,804 for women. These data are consistent with the 2010 Health Survey and the 2011 DARIOS study, which reported that the prevalence of DM-2 was higher among men than women. However, the mean values did not differ significantly between the sexes. This finding suggests that hospital treatment for DF ulcers is equally complex for male and female patients.

The analysis of hospital stays by age group showed that patients aged 75–79 years, who produced the most admissions (364), had an average stay of 10.6 days. This group also produced the highest total expenditure, €2,721,309, but the average cost of €7476 was not significantly different from that produced by the other age groups. Patients aged over 60 years present greater comorbidity, which complicates the hospitalisation situation [6], as does the very long duration of the disease (55 years, according to the 2009 European Health Survey). According to one study, 11% of all health expenditure is dedicated to treating DM-2 and 75% of this is provided to patients aged 50–79 years [7]. Our own findings confirm these results.

The youngest group of patients, aged 20–24 years, contained only two cases, with a mean hospital stay of 29.5 days. Hence, younger patients do not commonly require hospital admission, but when they do, the severity of the process means that hospitalisation can be prolonged. In contrast, the oldest patients, aged over 90 years, have an average stay of 5–6 days, although this could be because they present other, more serious, pathologies and therefore are derived to medium–long-stay institutions. Due to the small number of cases in these extreme groups, the youngest and oldest age groups were combined in our analysis.

Our cost analysis showed that a total hospital cost of €20,624,337 was incurred by these patients, an average of €7632 each. In Europe, the 2008 Eurodiale study estimated the average cost per patient to be between €7147 and €18,790, according to the complexity of the case. However, this figure increases to €24,540 if the patient is treated by amputation [11]. In the USA, corresponding values of $5500 and $28,000 per patient per year have been reported [10]. Our results corroborate those of Habacher [12], who estimated the basic treatment cost for diabetic ulcers to be €1171 for outpatients, rising to €7844 for more complex cases requiring hospitalisation.

### Clinical Implications and Study Limitations

Diabetes presents high morbidity, with an estimated prevalence in Spain of 6–13%. However, very little research has been conducted into the costs of hospitalisation in this country for patients with DF. Our results provide an up-to-date, cost-centred view of this question. The findings of the present study may provide useful guidance for the implementation of hospital treatment protocols by multidisciplinary teams.

The study presents certain limitations. Firstly, the analysis only considered hospital admissions for DF ulcers treated in public hospitals in the region of Valencia, during a relatively short period. No consideration was given to other situations, such as long-stay, chronic patients, private hospitals, primary care, day centres or nursing homes. The inclusion of a larger variety of data sources would provide a more accurate idea of the magnitude of the problem. In addition, our study design may be subject to sampling bias, as only patients’ gender and age were taken into consideration. Although data for comorbidities are provided, we were unable to evaluate their cost with respect to treatment for DF.

## 5. Conclusions

The total hospital cost of treatment for patients with DF ulcers during the five-year study period was €20,624,337, with an average of €7633 per admission. This average cost is below that reported by most previous studies. Male patients produced a higher total cost than females, but the mean cost per admission did not vary significantly between the sexes. By age group, the patients aged 75–79 years had the highest total cost, while those aged over 90 years presented the lowest total cost. The total hospital stay for these patients was 30,886 days. The mean stay was 11.4 days, which is consistent with previous results for Spain. The longest mean hospital stay (12.6 days) was recorded for 2009, and the shortest (10.1 days) for 2012. By gender, the total length of stay by male patients was 21,163 days vs. 9723 days for females. The average hospital stay was 11.3 days for men and 11.7 days for women. By age group, the longest average stay was presented by the patients aged 20–24 years, with 29.5 days, and the shortest by those aged 90–94 years, with 5.6 days.

## Figures and Tables

**Table 1:** Total annual treatment cost in public hospitals in Valencia.

Year	Number	Average Length of Hospital Stay (days)	Total €	Average €	SD	Standard Error	*p*-Value
2009	554	12.6	4,253,373.56	7677.57	4293.24	182.40	0.524
2010	554	12.3	4,130,284.55	7455.39	4223.43	179.44	
2011	556	11.6	3,773,626.11	7313.23	3794.65	167.05	
2012	500	10.1	3,818,912.68	7637.83	4198.12	187.75	
2013	578	105	4,648,140.39	8041.77	4321.33	179.74	
Total	2702	11.4	20,624,337.29	7632.99	4180.75	80.43	

**Table 2:** Health care admissions by DRG.

DRG	N	%	Cumulative %
LOWER LIMB AMPUTATION DUE TO ENDOCRINE, NUTRITIONAL AND METABOLIC DISORDERS	417	15.4	15.4
DIABETES AGE > 35	373	13.8	29.2
PERIPHERAL VASCULAR DISORDERS WITH COMPLICATIONS	320	11.8	41.1
AMPUTATION OF UPPER LIMB OR TOES DUE TO CIRCULATORY DISORDERS	253	9.4	50.4
SKIN ULCERS	209	7.7	58.2
AMPUTATION DUE TO CIRCULATORY DISORDERS EXCEPT UPPER LIMB OR TOES	167	6.2	64.4
SKIN GRAFT AND WOUND DEBRIDEMENT DUE TO ENDOCRINE, NUTRITIONAL AND METABOLIC DISORDERS	142	5.3	69.6
PROCEDURE ON CRANIAL AND PERIPHERAL NERVES AND OTHER SURGICAL PROCESSES NERVOUS SYSTEM WITH COMPLICATIONS	107	4.0	73.6
DISORDERS OF CRANIAL AND PERIPHERAL NERVES WITH COMPLICATIONS	100	3.7	77.3
OTHER SURGICAL PROCEDURES OF THE CIRCULATORY SYSTEM	87	3.2	80.5
OTHER VASCULAR PROCEDURES WITH COMPLICATIONS	75	2.8	83.3
OTHER VASCULAR PROCEDURES WITH GREATER COMPLICATIONS	47	1.7	85
MAJOR CARDIOVASCULAR PROCEDURES WITH COMPLICATIONS	41	1.5	86.5
SKIN GRAFT AND/OR DEBRIDEMENT DUE TO SKIN ULCER, CELLULITIS WITH COMPLICATIONS	38	1.4	87.9
OTHER ENDOCRINE, NUTRITIONAL AND METABOLIC SURGICAL PROCEDURES WITH COMPLICATIONS	38	1.4	89.3
MAJOR SKIN AND BREAST DISORDERS WITH MAJOR COMPLICATIONS	37	1.4	90.7
CIRCULATORY DISORDERS EXCEPT ACUTE MYOCARDIAL INFARCTION, ENDOCARDITIS, ICC AND ARRHYTHMIA WITH MAJOR COMPLICATIONS	36	1.3	92.0

**Table 3:** Cost by sex.

Gender	Patients (n)	Total €	Average €	SD	Standard Error	*p*-Value
**Male**	1868	14,386,532.37	7701.57	4089.59	94.62	0.202
**Female**	834	6,237,804.93	7479.38	4376.66	15.55	

**Table 4:** Cost by sex and age group.

Gender	Age	N	Total €	Average €	SD	Standard Error
Male	00–39	39	264,823.31	6790.34	3550.59	568.55
	40–44	72	515,803. 37	7163.94	3146.74	370.85
	45–49	116	851,428.83	7339.90	3894.44	361.59
	50–54	224	1,768,791.93	7896.39	3814.54	254.87
	55–59	226	1,818,751.65	8047.57	4114.49	273.69
	60–64	264	1,926,964.68	7299.11	3797.52	233.72
	65–69	257	2,045,970.79	7960.98	4291.12	267.67
	70–74	225	1,744,595.33	7753.76	4457.39	297.16
	75–79	215	1,627,810.19	7571.21	4034.32	275.14
	80–84	144	1,120,938.26	7784.29	4097.61	341.47
	85–89	68	573,845.44	8438.90	4978.98	603.79
	≥0	18	126,808.60	7044.92	5577.93	1314.73
Female	00–39	9	57,448.81	6383.20	2473.36	824.45
	40–44	13	82,486.45	6345.11	3067.76	850.84
	45–49	34	234,820.21	6906.48	4762.78	816.81
	50–54	53	381,040.48	7189.44	5426.94	745.45
	55–59	56	453,994.97	8107.05	3758.55	502.26
	60–64	75	606,804.67	8090.73	4134.75	477.44
	65–69	74	555,832.53	7511.25	5025.85	584.24
	70–74	93	719,135.19	7732.64	4955.88	513.90
	75–79	149	1,093,499.15	7338.92	3552.07	291.00
	80–84	150	1,096,036.81	7306.91	4515.66	368.70
	85–89	101	769,642.75	7620.23	4143.77	412.32
	≥90	27	187,062.91	6928.26	4918.99	946.66

## Abbreviations

MBDS	(Minimum Basic Data Set)
DF	(diabetic foot)
DM	(Diabetes mellitus)
DRG	(Diagnostic related group)
MDG	(Major diagnostic groups)
SD	(Standard deviation)
€	(euro)
